# Mitochondrial Dysfunction Is an Early Consequence of Partial or Complete Dystrophin Loss in *mdx* Mice

**DOI:** 10.3389/fphys.2020.00690

**Published:** 2020-06-19

**Authors:** Timothy M. Moore, Amanda J. Lin, Alexander R. Strumwasser, Kevin Cory, Kate Whitney, Theodore Ho, Timothy Ho, Joseph L. Lee, Daniel H. Rucker, Christina Q. Nguyen, Aidan Yackly, Sushil K. Mahata, Jonathan Wanagat, Linsey Stiles, Lorraine P. Turcotte, Rachelle H. Crosbie, Zhenqi Zhou

**Affiliations:** ^1^Department of Biological Sciences, Dana & David Dornsife College of Letters, Arts, and Sciences, University of Southern California, Los Angeles, CA, United States; ^2^Division of Endocrinology, Diabetes, and Hypertension, David Geffen School of Medicine, University of California, Los Angeles, Los Angeles, CA, United States; ^3^VA San Diego Healthcare System, San Diego, CA, United States; ^4^Department of Medicine, University of California, San Diego, La Jolla, CA, United States; ^5^Division of Geriatrics, Department of Medicine, University of California, Los Angeles, Los Angeles, CA, United States; ^6^Department of Integrative Biology and Physiology, University of California, Los Angeles, Los Angeles, CA, United States; ^7^Department of Neurology, David Geffen School of Medicine, University of California, Los Angeles, Los Angeles, CA, United States; ^8^Molecular Biology Institute, University of California, Los Angeles, Los Angeles, CA, United States

**Keywords:** muscular dystrophy, Duchenne muscular dystrophy, skeletal muscle, dystrophin, metabolism, mitochondria, autophagy

## Abstract

Duchenne muscular dystrophy (DMD) is characterized by rapid wasting of skeletal muscle. Mitochondrial dysfunction is a well-known pathological feature of DMD. However, whether mitochondrial dysfunction occurs before muscle fiber damage in DMD pathology is not well known. Furthermore, the impact upon heterozygous female *mdx* carriers (*mdx*/+), who display dystrophin mosaicism, has received little attention. We hypothesized that dystrophin deletion leads to mitochondrial dysfunction, and that this may occur before myofiber necrosis. As a secondary complication to mitochondrial dysfunction, we also hypothesized metabolic abnormalities prior to the onset of muscle damage. In this study, we detected aberrant mitochondrial morphology, reduced cristae number, and large mitochondrial vacuoles from both male and female *mdx* mice prior to the onset of muscle damage. Furthermore, we systematically characterized mitochondria during disease progression starting before the onset of muscle damage, noting additional changes in mitochondrial DNA copy number and regulators of mitochondrial size. We further detected mild metabolic and mitochondrial impairments in female *mdx* carrier mice that were exacerbated with high-fat diet feeding. Lastly, inhibition of the strong autophagic program observed in adolescent *mdx* male mice via administration of the autophagy inhibitor leupeptin did not improve skeletal muscle pathology. These results are in line with previous data and suggest that before the onset of myofiber necrosis, mitochondrial and metabolic abnormalities are present within the *mdx* mouse.

## Introduction

Muscular dystrophies are a family of genetic disorders manifesting primarily by the progressive wasting of skeletal muscle. Duchenne muscular dystrophy (DMD) is the most severe and frequent muscular dystrophy with most patients having little, if any, detectable dystrophin within muscle (Monaco et al., 1988; Emery, 1989; Govoni et al., 2013; De Palma et al., 2014). DMD patients present with clinical manifestations early in life and experience progressively deteriorating muscles until eventual passing before age 30 (Davies et al., 1988; Pichavant et al., 2011). Currently, DMD treatments delay disease progression or mitigate its symptoms, but frequently produce adverse side effects (Muntoni et al., 2002; Dubowitz, 2005; Bushby et al., 2010a, b). Additional developing therapies hold promise, yet many challenges lay ahead with such treatments likely several years from the accepted standard of care and FDA approval (Marshall and Crosbie-Watson, 2013; Young et al., 2016; Jones, 2019). Therefore, there is a need for alternative strategies, including combination-based therapies, to be developed from a more complete understanding of the cellular and physiological impact of dystrophin loss upon the myofiber. The most commonly used mouse model to study DMD is the *mdx* mouse (Bulfield et al., 1984). Although *mdx* muscles share some histological features with DMD, the phenotype is less severe, particularly concerning the associated cardiomyopathy and respiratory dysfunction that is life-threatening in DMD (McIntosh et al., 1998a, b).

Recent studies indicate mitochondria can adapt in size and morphology to changes in the cellular environment in virtually all cell types assessed (Liesa et al., 2009; Twig and Shirihai, 2011; Lackner, 2014). Dysfunction of this adaptive response can lead to dysmorphology, impaired oxidative phosphorylation, metabolic dysfunction, and an inability to adapt to stressors (Taanman, 1999; Bach et al., 2003; Miller et al., 2003; Twig et al., 2008; Nochez et al., 2009; Chen et al., 2010; Jornayvaz and Shulman, 2010; Pejznochova et al., 2010; Seo et al., 2010; Westermann, 2010; Scarpulla, 2011; Chan, 2012; Dickinson et al., 2013; Shen et al., 2014; Montgomery and Turner, 2015). Evidence links muscular dystrophies with mitochondrial and metabolic dysfunction (Lucas-Héron et al., 1990; Kemp et al., 1993; Even et al., 1994; Mokhtarian et al., 1996; Sperl et al., 1997; Kuznetsov et al., 1998; McIntosh et al., 1998a; Cole et al., 2002; Angelin et al., 2007; Khairallah et al., 2007; Gulston et al., 2008; De Palma et al., 2012; Pauly et al., 2012). However, the timing of these defects with respect to DMD and *mdx* pathology is unknown. We sought to determine the impact of the loss of dystrophin on mitochondrial and metabolic dysfunction in both male and female *mdx* mice. We hypothesized that, due to the highly structured intracellular environment of muscle, lacking a structural protein (dystrophin) would lead to an aberrant mitochondrial and metabolic phenotype prior to myofiber necrosis. Our results indicate a mitochondrial and metabolic phenotype in both male and female *mdx* mice prior to the onset of muscle fiber abnormalities, potentially suggesting an early mitochondrial role in the etiology of this disease.

## Materials and Methods

### Ethical Approval

The University of California, Los Angeles Institutional Animal Care and Use Committee approved this study. All animal care, maintenance, surgeries, and euthanasia were conducted in accordance with this Institutional Animal Care and Use Committee and the National Institutes of Health.

### Animals

Jackson Laboratories (Bar Harbor, ME, United States) 001801 (genotype: C57BL/10ScSn-*Dmd^mdx^/J*) homozygous female laboratory mice were purchased and crossed with the recommended Jackson 000476 (genotype: C57BL/10ScSnJ) mice (Control) to generate hemizygous male (*mdx*) and female (*mdx* carrier) mice used for all studies. Mice were group-housed two to four per cage, fed chow diet *ad libitum* (8604, Teklad, calories: 25% protein, 14% fat, 54% carbohydrate) or high-fat diet (D12451, Research Diets, Inc., calories: 45% fat, 20% protein, 35% carbohydrates) *ad libitum* for 8 weeks where indicated, and on a 12-h light/dark cycle. Mice were fasted for 6 h prior to euthanasia. LPT (leupeptin) injections were given at 12 mg/kg every other day for 5 weeks, where indicated in 9-week-old mice.

### Glucose and Insulin Tolerance Tests

Glucose and insulin tolerance tests (GTT or ITT) were performed following a 6 h fast as previously described (Ribas et al., 2016). Briefly, the GTT consisted of an intraperitoneal dextrose (1 g/kg) injection and glucose was assessed at 15-min intervals over the 120-min testing period. The ITT consisted of an intraperitoneal insulin injection (0.7 U/kg). Blood samples were drawn, and glucose was measured at 0, 15, 30, 45, 60, 90, and 120 min post-injection.

### Plasma Analysis

Immediately following euthanasia, whole blood was removed via 27-gauge needle from the abdominal aorta and centrifuged at 2,000 × *g* for 2 min in EDTA-coated tubes. Plasma was analyzed for insulin and leptin using the Meso Scale Discovery (Rockville, MD, United States) platform following the manufacturer’s recommended protocol. Assessment of plasma triglyceride was determined using the L-Type TG M Assay and Cholesterol E (Wako Diagnostics, Mountain View, CA, United States). Assessment of plasma glucose was determined using HemoCue Glucose 201 Systems glucometer. Assessment of plasma creatine kinase-MB (CKMB) was determined using mouse CKMB ELISA kit (LS-F5745-1, LSBio, WA, United States).

### *Ex vivo* Skeletal Muscle Glucose Uptake

Whole-muscle *ex vivo* glucose uptake was assessed using 2-deoxyglucose uptake assay (Ribas et al., 2016). Briefly, soleus muscles were carefully excised from anesthetized animals and immediately incubated for 30 min in complete Krebs-Henseleit buffer with or without insulin (60 μU/ml) at 35°C. Muscles were then transferred to the same buffer containing [^3^H] 2-deoxyglucose (3 μCi/ml) and [^14^C] mannitol (0.053 μCi/ml), and incubated for 20 min before being blotted of excess liquid and frozen in liquid nitrogen. Muscles were homogenized in lysis buffer and counted for radioactivity. Glucose uptake was standardized to the non-specific uptake of mannitol and estimated as micromole of glucose uptake per gram of tissue.

### Grip Strength, Maximal Running Speed, and Dynamic Hanging

The following experiments were performed as previously described without variation (Moore et al., 2019). Mouse genotypes were blinded to the experimenter for all tests. Grip strength was assessed using the GT3 Grip Strength Meter (BIOSEB, Pinellas Park, FL, United States). Each mouse performed five trials and the highest three trials were averaged. Maximal running speed was assessed as described previously (Lerman et al., 2002). Mice were acclimated to the running treadmill on two separate occasions prior to the maximal running speed test. On testing day, mice performed a 5-min warm-up at 5–10 m/min. Treadmill speed was increased by 3 m/min until mice were unable to maintain the speed for 10 consecutive seconds with gentle encouragement. Mice were given three attempts at each speed and approximately 60 s of rest after each increase in treadmill speed. Dynamic hanging as assessed by latency to fall test, an index of grip strength and muscle endurance, was performed as previously described (Mandillo et al., 2014). Mice were acclimated to the wire grid on two separate occasions prior to testing. Mice performed three trials and the data were averaged and reported as a Mean ± SEM. Mice were given 5 min of rest between each trial.

### Nucleic Acid Extraction, cDNA Synthesis, and Quantitative RT-PCR

DNA and RNA were extracted from a portion of pulverized frozen muscle using DNeasy/RNeasy Isolation kits (Qiagen, Germantown, MD, United States) as described by the manufacturer. Isolated DNA and RNA were tested for concentration and purity using a NanoDrop Spectrophotometer (Thermo Scientific, Waltham, MA, United States). Isolated RNA was converted into cDNA, assessed for purity, and qPCR of the resulting cDNA levels was performed as previously described (Drew et al., 2014). All genes were normalized to the housekeeping genes Ppia or 18S. Mitochondrial DNA content was assessed as a ratio of mitochondrial DNA (mtCO2) to nuclear DNA (18S). Primers used for qPCR can be found in Supplementary Table S1.

### Immunoblot Analyses

Pulverized frozen muscle was used for immunoblotting. Proteins from each individual whole cell homogenate were normalized (expressed relative to the pixel densitometry) to glyceraldehyde 3-phosphate dehydrogenase (GAPDH, AM4300, Ambion, Foster City, CA, United States). Phosphorylation-specific proteins were normalized (expressed relative to pixel densitometry) to the same unphosphorylated protein (i.e., phosphorylated Drp1 at Ser 616 was expressed relative to the pixel densitometry of Drp1 for each individual sample). See Supplementary Table S2 for a list of the primary antibodies used.

### Mitochondrial Isolation

Mitochondria were isolated from fresh gastrocnemius muscles using Mitochondria Isolation Kit (Thermo Scientific, Waltham, MA, United States) via Dounce homogenization for Hard Tissue protocol (Zhang et al., 2010; Benador et al., 2018). Briefly, 70 mg of fresh gastrocnemius muscles were rinsed by cold PBS twice. Then muscles were quickly minced, dounced, and centrifuged at 700 × *g* for 10 min at 4°C to discard tissue debris and nucleus. The supernatant was further centrifuged at 12,000 × *g* for 15 min at 4°C to acquire mitochondrial pellets. Subsequent immunoblotting underwent the same procedure described in the “Immunoblot Analyses” section.

### Tissue Histology

Tibialis anterior or gastrocnemius muscles were sectioned and stained for HE, SDH, and COX as previously described (Wanagat et al., 2001). Semi-quantitative analyses were performed on a blind basis using a scale (high, medium, and low density of staining) for each slide by three individuals.

### Transmission Electron Microscopy (TEM)

Soleus muscle was quickly and carefully excised from the lower hindlimb. Specifically, a small incision was made perpendicular to the distal end of the Achilles tendon. While cautiously preserving the underlying tissue, the skin was cut to expose the lower hindlimb muscle. The Achilles tendon was then cut and the entire muscle group consisting of the gastrocnemius, plantaris, and soleus was meticulously rotated uncovering the soleus muscle underneath. Great care was taken to ensure the muscle was not stretched or distorted. Using a surgical scalpel, small cuts were made around the visible soleus muscle removing it from the surrounding tissue. The soleus was examined for any remaining tissue which was removed if present and then immersed in freshly prepared fixative containing 2.5% glutaraldehyde and 2% paraformaldehyde in 0.15 M cacodylate buffer and stored at 4°C until use as described previously (Doughty et al., 1995; Park et al., 2016). This fixative has been shown to preserve muscle architecture. After fixation, muscles were processed for TEM analysis as described previously (Zhou et al., 2018). Ultrathin (∼60 nm) sections were viewed using a JEOL 1200EX II (JEOL, Peabody, MA, United States) electron microscope and photographed using a Gatan digital camera (Gatan, Pleasanton, CA, United States) as previously described. Mitochondrial area, perimeter, Feret’s diameter, and cristae numbers were analyzed and quantified in all images by three separate and blinded individuals using ImageJ (NIH).

### Complex IV Enzyme Activity Assay

Mitochondrial complex IV enzymatic activity was measured as instructed (Complex IV Rodent Enzyme Activity Microplate Assay Kit, Abcam, ab109911). Briefly, same amounts of homogenized muscle sample were loaded on the plate and incubated at room temperature for 3 h. After rinsing wells twice with solution 1, the plate was read at OD550 with assay solution. Complex IV activity was determined by calculating the slope between two points within the linear region.

### Mitochondrial Respirometry

Frozen skeletal muscle tissues were thawed on ice and homogenized in MAS (70 mM sucrose, 220 mM mannitol, 5 mM KH2PO4, 5 mM MgCl2, 1 mM EGTA, 2 mM HEPES, pH 7.4). The samples were mechanically homogenized with 60 strokes in a teflon-glass dounce homogenizer. All homogenates were centrifuged at 1000 × *g* for 10 min at 4°C then the supernatant was collected. Protein concentration was determined by BCA (Thermo Scientific, Waltham, MA, United States). Homogenates were loaded into Seahorse XF96 microplate in 20 μL of MAS at 6 μg/well. The loaded plate was centrifuged at 2,400 × *g* for 10 min at 4°C (no brake) and an additional 130 μL of MAS supplemented with 100 μg/mL cytochrome c was added to each well. Substrate injection were as follows: Port A: NADH (1 mM) or succinate + rotenone (5 mM + 2 μM); Port B: rotenone + antimycin A (2 μM + 2 μM); Port C: *N*,*N*,*N*′,*N*′-tetramethyl-p-phenylenediamine (TMPD) + ascorbic acid (0.5 mM + 1 mM); and Port D: azide (50 mM). These conditions allow for the determination of the maximal respiratory capacity of mitochondria through Complex I, Complex II, and Complex IV.

### Statistical Analysis

Values are presented as mean ± SEM and expressed relative to the average value obtained for each experimental control group unless otherwise stated. Statistical analyses were performed using Student’s *t*-test when comparing two groups of samples or one-way analysis of variance (ANOVA) with Tukey’s *post hoc* comparison for identification of significance within and between groups using GraphPad Prism 5 (GraphPad Software, San Diego, CA, United States). Significance was set *a priori* at *P* < 0.05.

## Results

### Regulators of the Mitochondrial Life Cycle Are Altered in Skeletal Muscle From 40-Week-Old, Male *mdx* Mice

**FIGURE 1:**
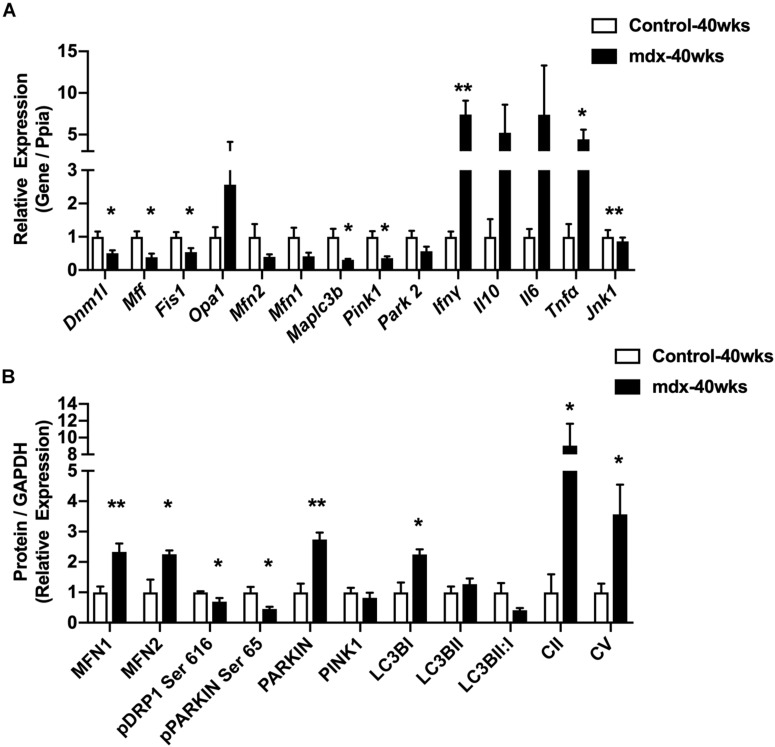
40-week-old *mdx* male muscles display altered regulators of the mitochondrial life cycle. **(A)** Gene expression from gastrocnemius muscle (*N* = 4–5). **(B)** Protein expression from gastrocnemius muscle (*N* = 4–5). Data presented as Mean ± SEM. *, **P < 0.05, 0.01, respectively.

To ascertain the impact of lacking dystrophin upon mitochondria, we quantified protein and RNA expression for several regulators of the mitochondrial network in gastrocnemius muscles from 40-week-old male mice (*mdx*-40wks), which represents the late, hypertrophic stage of disease. Gene expression for the inflammatory cytokines IFNγ, IL10, IL6, and TNFα were elevated by approximately 4.5 to 7.5-fold in *mdx* males compared with age-matched controls (Figure 1A). We also observed substantial reductions of genes related to mitochondrial fission (*Dnm1l*, *Mff*, and *Fis1*) and mitophagy (*Maplc3b* and *Pink1*) in *mdx* mice vs. age-matched controls (Figure 1A). Reduced phosphorylation of Drp1 at Serine 616, a pro mitochondrial fission signal, and elevated protein levels of mitochondrial fusion regulators (Mfn1 and Mfn2; Figure 1B and Supplementary Figure S1) were evident. Moreover, the protein levels of mitophagy related factors (Parkin and Lc3bI) were also elevated in *mdx* males compared with age-matched controls (Figure 1B and Supplementary Figure S1). In general, we found regulators of mitochondrial life cycle are altered in the skeletal muscle from 40-week-old, male *mdx* mice.

### Dysfunction of Mitochondrial Enzymatic Activity and Morphology Are Evident at Early Stages of Dystrophin-Deficient Disease

**FIGURE 2:**
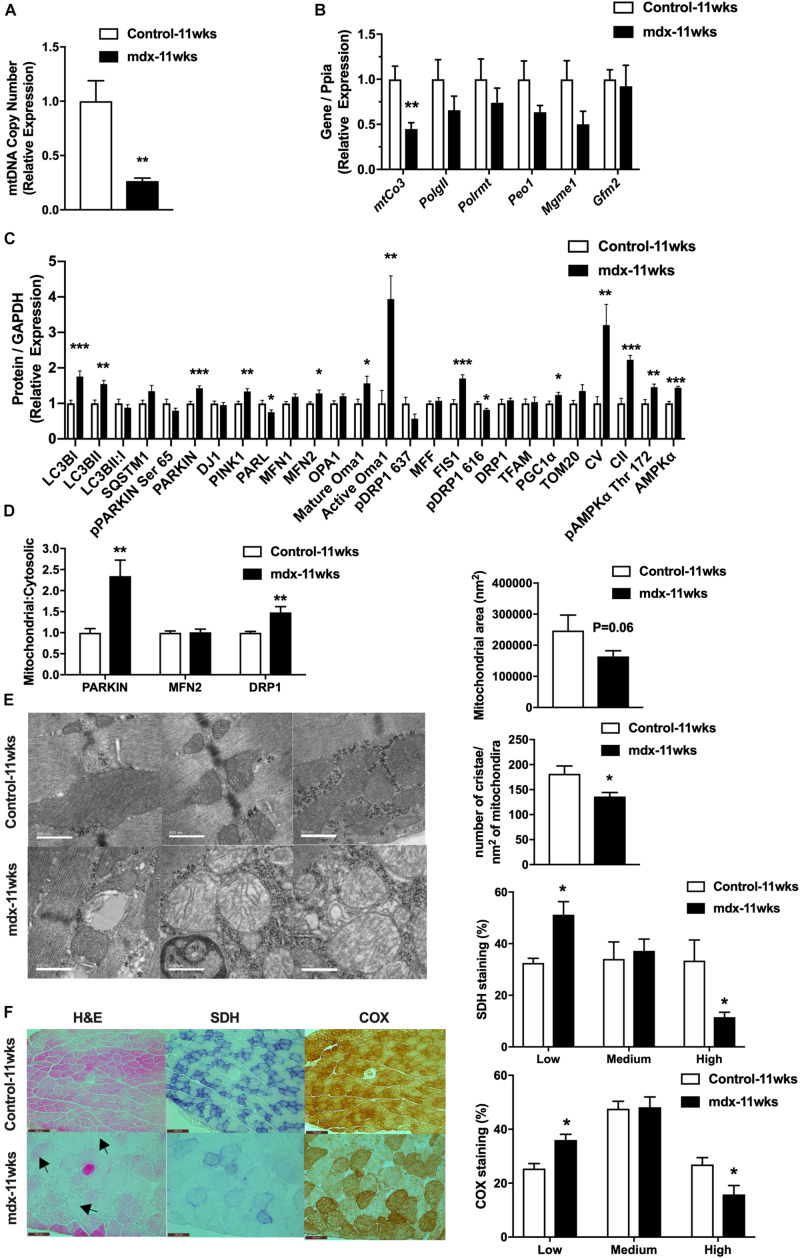
11-week-old, *mdx* male muscles display altered regulators of the mitochondrial life cycle, enzymatic activity, and mitochondrial shape. **(A)** Mitochondrial DNA copy number in quadriceps muscle (*N* = 6–8). **(B)** mRNA expression in quadriceps muscle (*N* = 5–8). **(C)** Protein expression in quadriceps muscle (*N* = 6–8). **(D)** Protein expression in mitochondria isolated from quadriceps muscle (*N* = 6–8). **(E)** Electron micrograph images of the soleus muscle with quantified mitochondrial area and cristae numbers per area of mitochondria shown right. **(F)** HE, SDH, and COX staining in tibialis anterior muscle with the percentage of muscle fiber density shown right (*N* = 13). Black arrow indicates fibers with centralized nuclei. Only some fibers are indicated. Scale bar = 0.1 mm. Data presented as Mean ± SEM. *, **, ***P < 0.05, 0.01, 0.001, respectively.

Because of the large changes in the expression of regulators of the mitochondrial network observed in late stage of disease (*mdx*-40wks), we hypothesized that these responses resulted from widespread muscle damage and chronic inflammation that eventually causes muscle fiber apoptosis and necrosis. To test this hypothesis, we investigated 11-week-old, *mdx* male mice (*mdx*-11wks), which represents the active regeneration phase of the disease (prior to the onset of severe muscle damage, apoptosis, and necrosis) to determine if mitochondrial network alterations occur because of or as a precursor to severe muscle damage as observed previously (Ieronimakis et al., 2016). We found that quadriceps muscle from *mdx*-11wks male mice display reduced mitochondrial DNA copy number as well as reduced mitochondrial DNA derived transcript (mtCO3) relative to age-matched controls (Figures 2A,B). We observed no changes in the gene expression of regulators of mitochondrial DNA replication (*PolGII*, *Peo1*, and *Mgme1*), mitochondrial RNA polymerase (*Polrmt*), and protein translation (*Gfm2*; Figure 2B). At the protein level, several regulators of the mitochondrial network were altered including Lc3bI, Parkin, Pink1, Parl, Mfn2, mature and active Oma1, Fis1, Pgc1α, and Ampkα compared with age-matched controls (Figure 2C and Supplementary Figure S2). Active form of Ampkα (Phosphorylated threonine (Thr) 172) and Drp1 [serine (Ser) 616] were also significantly altered (Figure 2C and Supplementary Figure S2). Since changes in protein levels observed in whole-cell lysates might not reflect proteins present within or on mitochondria, we immunoblotted for a select number of proteins in isolated mitochondria from the quadriceps muscle. Both Parkin and Drp1 were significantly increased, but no change of Mfn2 in the mitochondrial fraction from *mdx*-11wks vs. age-matched controls (Figure 2D and Supplementary Figure S2) was observed. Electron micrograph images from the soleus muscle depicted a highly altered mitochondria network that included aberrant structure and cristae numbers per area of mitochondria (Figure 2E). Muscle damage was also overtly visible via altered fiber and z-line orientation. As expected, centralized nuclei were abundant in myofibers from mdx samples, which is consistent with ongoing muscle degeneration and regeneration, a hallmark of DMD (Figure 2F). Such changes occur concomitantly with reduced percentage of muscle fibers with high density of SDH and COX staining indicative of reduced mitochondrial function (Figure 2F).

### Mitochondria DNA Copy Number Is Reduced at the Onset of Tissue Abnormalities in 4-Week-Old, *mdx* Male Muscles

Having observed aberrant mitochondria in *mdx* muscle as early as 11 weeks of age, we extended our investigation to 4-week-old, *mdx* male mice (*mdx*-4wks), which represents the early state of the disease, just as the first signs of muscle regeneration are evident. Similar to our previous results, we observed a reduction in mitochondrial DNA copy number in quadriceps muscles of *mdx*-4wks vs. age-matched control mice (Supplementary Figure S3A). Histological staining of gastrocnemius muscle from these same animals revealed muscle fibers with centralized nuclei, localized reductions in fiber cross-sectional area, and regions of nuclei accumulation potentially indicative of inflammatory cell infiltration (Supplementary Figure S3B). Therefore, even at 4 weeks of age, *mdx* muscle exhibits reduced mitochondrial DNA copy number simultaneously with the onset of muscle fiber damage.

### Mitochondria From Pre-necrotic 2-Week-Old, *mdx* Male Muscle Displays Altered Cristae Structure

**FIGURE 3:**
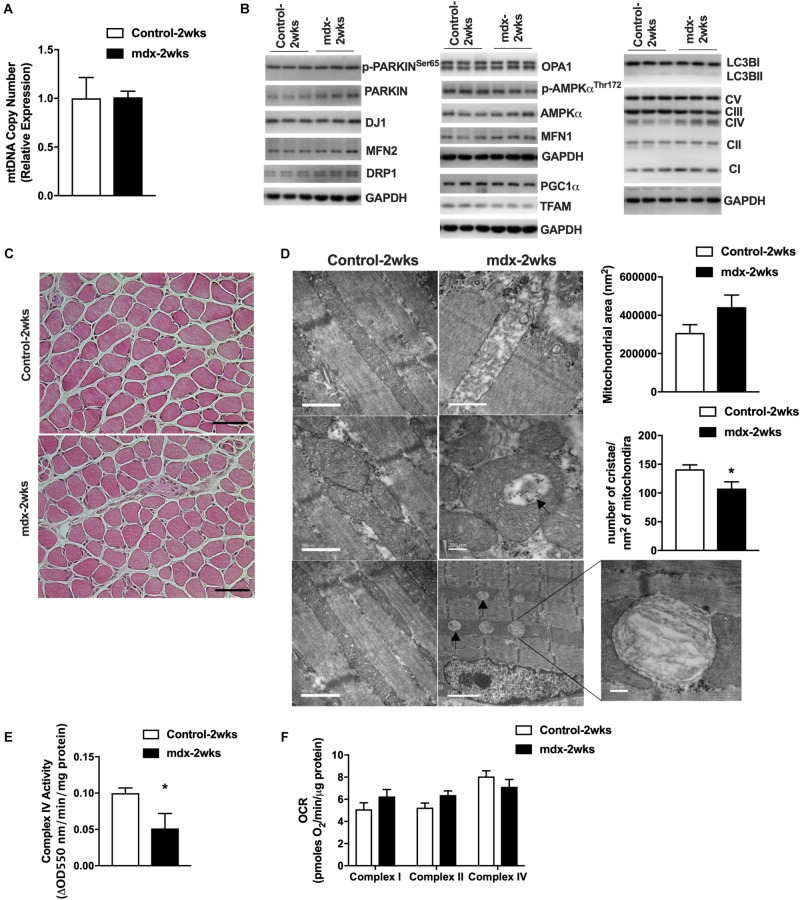
Mitochondria from 2-week-old, *mdx* male muscles display altered size, cristae numbers per area of mitochondria, and enzymatic activity. **(A)** Mitochondrial DNA copy number in quadriceps muscle (*N* = 5). **(B)** Immunoblot images from quadriceps muscle (*N* = 6–8, showing *N* = 3). **(C)** HE stain of gastrocnemius muscle. Scale bar = 0.1 mm. **(D)** Electron micrograph images of the soleus muscle with quantified mitochondrial area and cristae numbers per area of mitochondria shown right. Black arrows indicate aberrant mitochondria. **(E)** Complex IV activity in quadriceps muscle (*N* = 5). Data presented as Mean ± SEM. **(F)** Mitochondrial respirometry analysis in frozen quadriceps muscle (*N* = 6–8). *P < 0.05.

To further test our hypothesis regarding the connection between mitochondria and muscle damage, we generated 2-week-old, *mdx* male mice (*mdx*-2wks), which represent the pre-necrotic stage of disease and is before the onset of overt muscle pathology. We measured mitochondrial DNA copy number in quadriceps muscles and found no difference between *mdx*-2wks and age-matched controls (Figure 3A). Quadriceps muscles were immunoblotted for several proteins related to mitochondria, mitophagy, fission, fusion, and biogenesis which showed no differences in levels of these marker proteins between the two groups (Figure 3B). Muscle fiber morphology was examined by histological staining of the gastrocnemius muscle, revealing no signs of muscle damage (Figure 3C). Despite observing no differences in mitochondrial DNA copy number, protein levels, and muscle fiber morphology, we found reduced cristae numbers per area of mitochondria and the presence of mitochondrial vacuoles in muscle from 2-week-old, *mdx* male mice in electron micrographs (Figure 3D and Supplementary Figure S4). Additionally, although mitochondrial oxygen consumption rate of complex I/II/IV were not altered, mitochondrial complex IV enzymatic activity was significantly decreased (Figures 3E,F). These results suggest the alteration of mitochondrial architecture and enzyme activity before the onset of muscle damage.

### Autophagy Inhibition Did Not Improve Muscle or Mitochondrial Phenotype in *mdx* Male Mice

**FIGURE 4:**
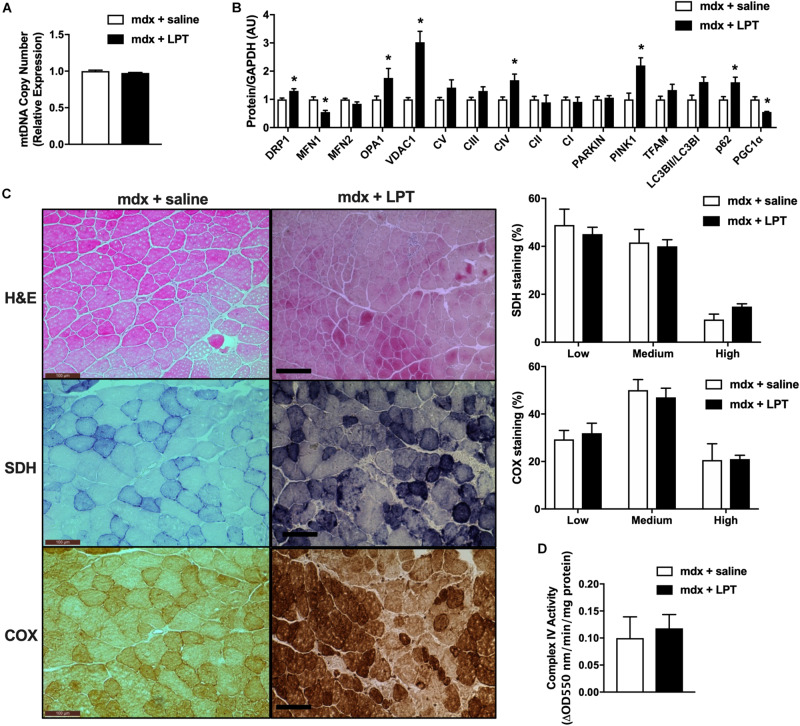
Autophagy inhibition did not improve mitochondrial phenotype in 14-week-old *mdx* male mice. **(A)** Mitochondrial DNA copy number in quadriceps muscle (*N* = 5). **(B)** Protein expression in quadriceps muscle (*N* = 5). **(C)** HE, COX, and SDH staining in tibialis anterior muscle with the percentage of muscle fiber density shown right (*N* = 12). Scale bar = 0.1 mm. **(D)** Complex IV activity in quadriceps muscle (*N* = 5). Data presented as Mean ± SEM. *P < 0.05.

Our previous results in *mdx* mice at 40 and 11 weeks of age revealed elevated levels of genes and proteins related to mitophagy and autophagy. Furthermore, electron micrograph images of *mdx*-2wks displayed mitochondrial vacuoles. These observations and other results from the research community have supported the concept of autophagy inhibition as a potential therapy for muscle wasting conditions (Selsby et al., 2010; Childers et al., 2011; De Palma et al., 2012). Therefore, we treated *mdx* male mice via intraperitoneal injections of saline (*mdx* + saline) or the autophagy inhibitor, leupeptin (*mdx* + LPT) for 5 weeks, as described previously (Salminen, 1984; Haspel et al., 2011; Esteban-Martínez and Boya, 2015), starting at 9 weeks of age in an attempt to preserve mitochondrial degradation. Within quadriceps muscles, mitochondrial DNA copy number was not changed in *mdx* + LPT vs. saline-treated *mdx* mice (Figure 4A). Without changing gene expression, LPT treatment elevated the protein level of mitochondrial proteins (Drp1, Vdac1, Opa1, and Complex IV) and mitophagy related factors (Lc3bII/I, p62, and Pink1), validating robust autophagy inhibition by LPT (Figure 4B and Supplementary Figures S5A,B). However, mitochondrial enzymatic histochemical and activity analyses of the tibialis anterior muscle showed no differences in LPT treated animals suggesting LPT treatment did not ameliorate mitochondrial dysfunction in *mdx* mice (Figures 4C,D).

### Female Asymptomatic *mdx* Carriers Present With Diet-Induced Obesity and Insulin Resistance

**FIGURE 5:**
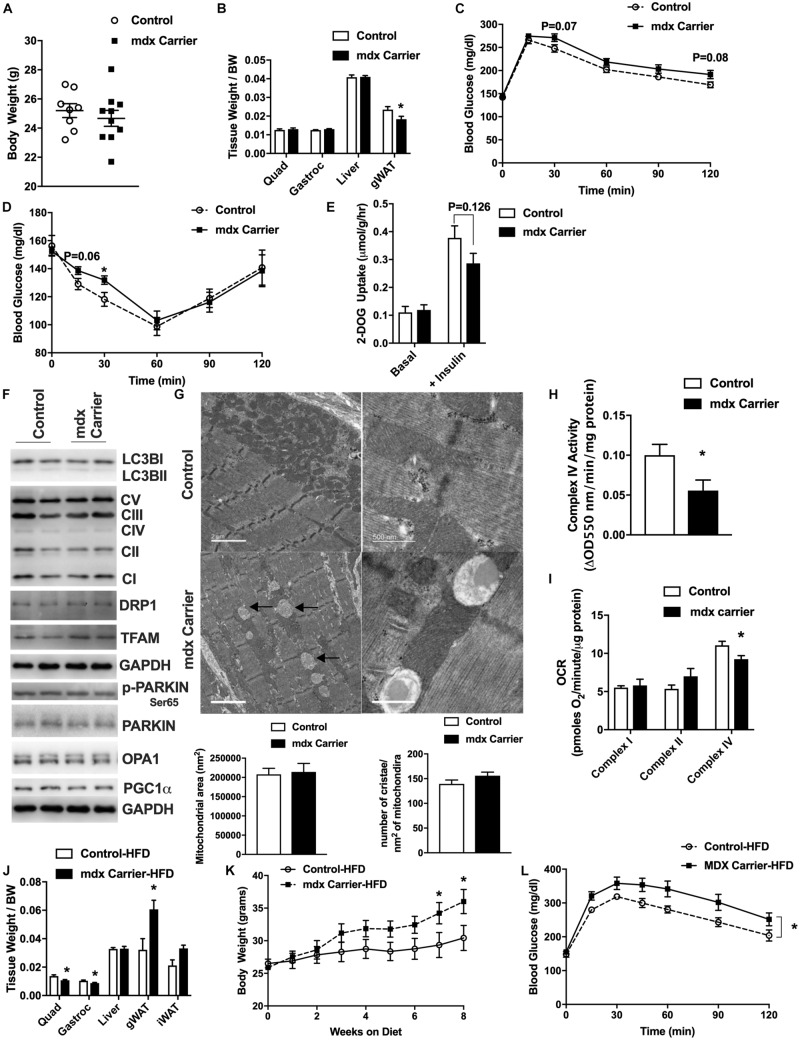
Female *mdx* carriers present mild metabolic impairments that are exacerbated with high-fat diet feeding. Female *mdx* carriers fed with normal chow **(A–I)** (*N* = 6–10): **(A)** Tissue weight relative to body weight. **(B)** Body weight (grams). **(C,D)** Glucose and insulin tolerance tests with area under the curve (AUC) insert. **(E)** 2-Deoxyglucose uptake with or without insulin in excised soleus muscle. **(F)** Immunoblot images for respective proteins. **(G)** Electron micrograph images of the soleus muscle with quantified mitochondrial area and cristae numbers per area of mitochondria shown bottom. Black arrow indicates mitochondrial vacuoles. **(H)** Complex IV activity in quadriceps muscle. **(I)** Mitochondrial respirometry analysis in quadriceps muscle. Female *mdx* carriers fed with high-fat diet **(J–L)** (*N* = 6–7): **(J)** Tissue weight relative to body weight. **(K)** Body weight during high-fat diet feeding. **(L)** Glucose tolerance tests with area under the curve (AUC) insert. Data presented as Mean ± SEM. *P < 0.05.

Women who are carriers of the DMD gene are largely asymptomatic with regard to skeletal muscle symptoms, but are susceptible to cardiomyopathy (Childers and Klaiman, 2017; Viggiano et al., 2017; Florian et al., 2018; Ishizaki et al., 2018; Zhong et al., 2019). Given our observation of mitochondrial defects in presymptomatic *mdx* male muscle, we sought to query the impact upon mitochondria, muscle, and metabolism in adult 24-week-old, female *mdx* carrier (*mdx* carriers) mice. Adult female *mdx* carriers were chosen because human female *mdx* carriers typically display symptoms during adult hood and because maximal muscle growth has nearly been achieved according to Jackson laboratory growth curves. We found that female *mdx* carriers displayed similar body weight to age-matched controls (Figure 5A). These mice showed a lower gonadal white adipose tissue mass (gWAT) although no differences in the weights of other metabolic organs (Figure 5B) were observed. Because of the role of muscle in glucose homeostasis, we performed glucose and insulin tolerance tests (GTT and ITT, respectively). Female *mdx* carrier mice showed slight impairments in glucose and insulin sensitivity although plasma insulin, leptin, and triglyceride values were not different compared to age-matched controls (Figures 5C,D and Supplementary Figures S6A–C). *Ex vivo* glucose uptake into excised soleus muscles revealed a similar modest reduction in insulin-stimulated glucose uptake in female *mdx* carriers, although this did not reach statistical significance (Figure 5E). We then performed functional muscle strength and endurance testing and found no differences between the two groups in these parameters (Supplementary Figures S6D–F). Our previous results indicated that a loss of dystrophin had an impact upon the expression of regulators of the mitochondria life cycle within skeletal muscle from male mice. However, female *mdx* carriers showed no differences in proteins related to mitophagy, autophagy, mitochondrial biogenesis, fission, or fusion (Figure 5F). Nevertheless, electron micrograph images from female *mdx* carriers consistently displayed mitochondrial vacuoles, similar to what was observed in presymptomatic 2-week-old, *mdx* male mice, despite no overt changes to the muscle fiber or z-line orientation (Figure 5G). Moreover, we performed both enzymatic and respirometry assays and observed a significant reduction of mitochondrial complex IV activity in female *mdx* carriers, suggesting a defect in mitochondrial function (Figures 5H,I). Interestingly, we found that high-fat diet (HFD) administration significantly reduced quadriceps and gastrocnemius muscle weights, dramatically elevated body weight, gWAT weight, impaired GTT, and elevated fat mass ratio without changing plasma triglyceride and lactate levels in female *mdx* carriers (Figures 5J–L and Supplementary Figures S6G–I). These results suggest female *mdx* carriers present with mild metabolic impairment that is exacerbated with HFD administration.

## Discussion

A greater understanding of the many consequences of loss of dystrophin are needed in order to inform the design of new therapies. Because of this, we sought to understand the biological impact of lacking functional dystrophin protein upon mitochondria and metabolism within male and female mice. Utilizing the *mdx* mouse, where males have little dystrophin expression and female carriers display dystrophin somatic mosaicism, we detected via electron microscopy, aberrant mitochondrial morphology, reduced cristae numbers, and large empty spaces within mitochondria in both male and female *mdx* mice prior to the onset of muscle fiber damage. To our knowledge, this work represents the first to suggest that mitochondrial ultrastructure is impacted prior to muscle fiber damage. We further observed impaired complex IV enzymatic activity as previously described (Gaglianone et al., 2019). While not extensively tested, published research does suggest a mitochondrial phenotype occurring early in muscular dystrophy disease progression (Nghiem et al., 2017; Vila et al., 2017; Barker et al., 2018). These data suggest that a connection may exist between mitochondria, dystrophin, and muscle fiber damage, at least in a mouse model of muscular dystrophy. Our results are also in agreement with data showing genetic or pharmacological increases in PGC1α, a known master regulator of mitochondria, improving recovery from injury in *mdx* mice (Jahnke et al., 2012; Selsby et al., 2012; Chan et al., 2014).

Male *mdx* mice have already been shown to possess metabolic abnormalities after disease onset and during progression (Blanchet et al., 2012; Strakova et al., 2018). In alignment with these results, we additionally observed mitochondrial dysfunction and metabolic disorder in female *mdx* carrier mice that was exacerbated with high-fat diet feeding. These findings indicate mitochondrial and metabolic dysfunction in female *mdx* carriers with increased susceptibility to diet-induced obesity and insulin resistance despite possessing one functional copy of the dystrophin allele. To our knowledge, this is the first characterization of metabolism and mitochondria within female *mdx* carrier mice. Phenotypic abnormalities, particularly related to mild muscle weakness and cramping, have been noted in a small subset of human female dystrophin mutation carriers (Hoffman et al., 1992; Ceulemans et al., 2008; Ameen and Robson, 2010; Walcher et al., 2010; Yoon et al., 2011; Brioschi et al., 2012; van Putten et al., 2012). Nevertheless, epidemiological evidence linking female dystrophin mutation carriers with increased prevalence of obesity, type 2 diabetes, or metabolic dysfunction is lacking. Furthermore, while such metabolic changes are minor in female *mdx* carrier mice, they do suggest that female humans harboring dystrophin mutations could be susceptible to metabolic dysfunction particularly in the context of diet-induced obesity or aging. This is also supported by altered glucose handling and metabolism in a mixed sex canine model of muscular dystrophy (Schneider et al., 2018). Female carriers of DMD may present with associated cardiomyopathy, which has led to more widespread cardiac monitoring of women with DMD offspring (Florian et al., 2016). Interestingly, mitophagy has been implicated in DMD cardiac disease (Kyrychenko et al., 2015; Kang et al., 2018), further supporting that mitochondria dysfunction is a common feature of DMD.

Despite the novelty of our findings, there are several limitations. Our main finding of mitochondrial vacuoles within and adjacent to mitochondria is inconclusive. These structures could represent swollen mitochondria due to calcium influx, enlarged lipid droplets, or autophagic vesicles. In addition, it has been postulated that due to the reduced structural integrity of skeletal muscle cells in the *mdx* mouse, artifacts may occur more easily during assays (Bereiter-Hahn and Vöth, 1979). Thus, they could simply represent actual empty spaces as a result of tissue handling. Therefore, further work is needed to verify the identity of these structures. In addition, despite the connection between autophagy, mitochondria, and muscular dystrophy (De Palma et al., 2012, 2014; Whitehead, 2016; Piras and Boido, 2018), autophagy inhibition did not improve disease pathology in *mdx* male mice, similar to previous results (Selsby et al., 2010; Childers et al., 2011; De Palma et al., 2012; Sandri et al., 2013). The severity of the disease at this age could preclude the ability of autophagy inhibition to reverse symptoms. Moreover, the failure of autophagy inhibition to improve mitochondrial or muscle fiber damage could be related to the relatively low dose administered (12 mg/kg), the method of administration which did not specifically target skeletal muscle (intraperitoneal injection), the infrequent dosing scheme employed (Q.O.D, every other day), or the short duration of administration (5 weeks). Finally, while we observed reductions in mtDNA, previous results indicate *mdx* muscle having reduced numbers of nuclei per muscle fiber, smaller muscle fibers, and an increase in non-muscle cells potentially biasing our results and causing incorrect conclusions to be made (Duddy et al., 2015). Despite observing no difference in nuclear DNA number between groups in all experiments, more robust methods are needed to verify the legitimacy of these data.

Collectively, our results substantiate previous findings and further expound upon mitochondrial and metabolic abnormalities prior to the onset of muscle damage in both male and female *mdx* mice. While the precise connection between dystrophin and mitochondria is still unknown, lacking functional dystrophin is believed to increase the permeability of the cell to Ca^2+^, promoting mitochondrial Ca^2+^ overload. Calcium overload leads to mitochondrial swelling, increased mitochondrial reactive oxygen species production, and mitochondrial permeability transition pore opening ultimately resulting in mitochondrial dysfunction (Millay et al., 2008; Perumal et al., 2015; Vila et al., 2017). In addition, because the sarcoendoplasmic reticulum (SR) physically interconnects with mitochondria within the muscle fiber and functions to store Ca^2+^, Ca^2+^ overload can also result in SR stress further contributing to mitochondrial dysfunction in *mdx* mice (Pauly et al., 2017). In conclusion, the research presented here, as well as from other groups, supports future endeavors to improve mitochondrial function as a component of combination-based therapies to combat muscular dystrophy.

## Conclusion

In line with previous data, prior to onset of muscle fiber damage, skeletal muscle from male *mdx* and female *mdx* carrier mice presented with aberrant mitochondrial structure, reduced cristae number, and large empty spaces within mitochondria in addition to reduced mitochondrial function. Moreover, female *mdx* carriers presented mild metabolic impairments that were exacerbated with high-fat diet feeding. These phenotypes prior to muscle damage suggest a mitochondrial component to the muscular dystrophy family of diseases. This insight can help shape future therapeutics to improve mitochondrial function and help mitigate the impact of this devastating group of diseases.

## Data Availability Statement

All datasets presented in this study are included in the article/Supplementary Material.

## Ethics Statement

The animal study was reviewed and approved by The University of California, Los Angeles Institutional Animal Care and Use Committee.

## Author Contributions

TM and ZZ performed the conception and design. TM, AL, AS, KC, KW, ThH, TiH, JL, DR, CN, AY, JW, SM, LT, LS, RC, and ZZ conducted the animal experiments, sample collection, and subsequent experimental analysis. TM and ZZ drafted the original manuscript. All authors contributed to the final drafting of the manuscript.

## Conflict of Interest

The authors declare that the research was conducted in the absence of any commercial or financial relationships that could be construed as a potential conflict of interest.

## Supplementary Material

FIGURE S1 Immunoblot images from 40-week-old gastrocnemius muscle.

FIGURE S2 Immunoblot images from 11-week-old quadriceps muscle.

FIGURE S3 Mitochondria DNA copy number is reduced at the onset of tissue abnormalities in 4-week-old *mdx* male muscles. **(A)** Mitochondrial DNA copy number in quadriceps muscle (*N* = 8–10). **(B)** HE stain of gastrocnemius muscle. Black arrows indicate fibers with centralized nuclei. Only some fibers are indicated. Scale bar = 0.1 mm. Data presented as Mean ± SEM. ^∗^*P* < 0.05.

FIGURE S4 Electron micrograph images of the soleus muscle from 2-week-old control and *mdx* male mice.

FIGURE S5 Immunoblot images and gene expression results from LPT treated quadriceps muscle. **(A)** Immunoblots of mitochondrial fission, fusion, mitophagy, and autophagy proteins in quadriceps muscle of *mdx* + saline and *mdx* + LPT (*N* = 5–6). **(B)** Gene expression of elevated protein targets in quadriceps muscle of *mdx* + saline and *mdx* + LPT (*N* = 5–6).

FIGURE S6 Plasma metabolites and muscle strength and endurance measurements. Plasma **(A)** insulin, **(B)** leptin, **(C)** triglyceride of Control and *mdx* carrier fed with normal chow (*N* = 5–10). **(D)** Maximum running speed, **(E)** latency to fall, **(F)** fore + hindlimb grip strength of control and *mdx* carrier fed with normal chow (*N* = 8–10). Plasma **(G)** triglyceride, **(H)** lactate, and **(I)** fat mass/body weight ratio of control and *mdx* carrier fed with HFD (*N* = 6–7). ^∗∗∗^*P* < 0.001.

TABLE S1 Primers used for qPCR.

TABLE S2 Antibodies used for immunoblotting.

## Abbreviations

18S: 18S ribosomal RNA
AMPKa: protein kinase AMPK-activated catalytic subunit alpha 1
AUC: area under the curve
CI: mitochondrial complex 1 NADH:ubiquinone oxidoreductase subunit B8
CII or II-30: mitochondrial complex 2 succinate dehydrogenase complex iron sulfur subunit B
CIII: mitochondrial complex 3 ubiquinol-cytochrome C reductase core protein II
CIV: mitochondrial encoded cytochrome C oxidase I
CV or V-a: mitochondrial complex 5 ATP synthase alpha
COX: cytochrome C oxidase
DJ1: Parkinson disease 7
DNM1L: see Drp1
Drp1: dynamin related protein 1
Esr1: estrogen receptor 1
Fis1, fission: mitochondrial 1
GAPDH: glyceraldehyde-3-phosphate dehydrogenase
Gastroc: gastrocnemius
Gfm2: G elongation factor mitochondrial 2
gWAT: gonadal white adipose tissue
HE: hematoxylin and eosin
HSPA1A: heat shock protein family A member 1A
HSPA1B: heat shock protein family A member 1B
IFNy: interferon gamma
IL10: interleukin 10
IL6: interleukin 6
JNK1: mitogen-activated protein kinase 8
LC3B: see MAPLC3B
LPT: leupeptin
MAPK1: mitogen-activated protein kinase 1
MAPLC3B: microtubule associated protein 1 light chain 3 beta
*mdx*: Duchenne muscular dystrophy mouse model
MFF: mitochondrial fission factor
MFN1: mitofusin 1
MFN2: mitofusin 2
Mgme1: mitochondrial genome maintenance exonuclease 1
mtCO3: cytochrome C oxidase subunit III
mtDNA: mitochondrial DNA
Oma1: overlapping with the M-AAA protease 1
Opa1: optic atrophy protein 1
Parl: presenilin associated rhomboid like
Park2: Parkin RBR E3 ubiquitin protein ligase
Peo1: twinkle mtDNA helicase
PGC1a: PPARG coactivator 1 alpha
Pink1: PTEN induced putative kinase 1
PolGII, DNA polymerase gamma 2: accessory subunit
Polrmt: RNA polymerase mitochondrial
Quad: quadriceps
Ser: serine
SDH: succinate dehydrogenase
SQSTM1: sequestosome 1
TFAM, transcription factor A: mitochondrial
Thr: threonine
Tom20: translocase of outer mitochondrial member
TNFa: tumor necrosis factor alpha
wks: weeks.
