# Supplementary figures and images for: Mitochondrial Dysfunction Is an Early Consequence of Partial or Complete Dystrophin Loss in mdx Mice
Source: Front Physiol. 2020 Jun 19;11:690. doi: 10.3389/fphys.2020.00690 (PMC7317021; doi:10.3389/fphys.2020.00690)

**FIGURE S1**

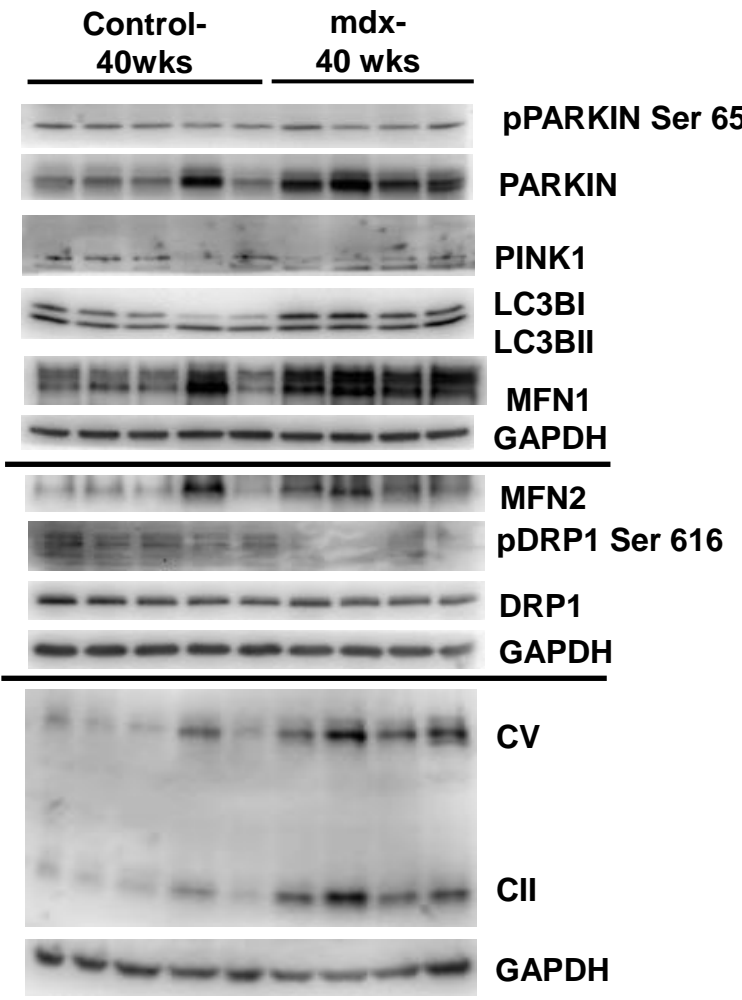

FIGURE S2

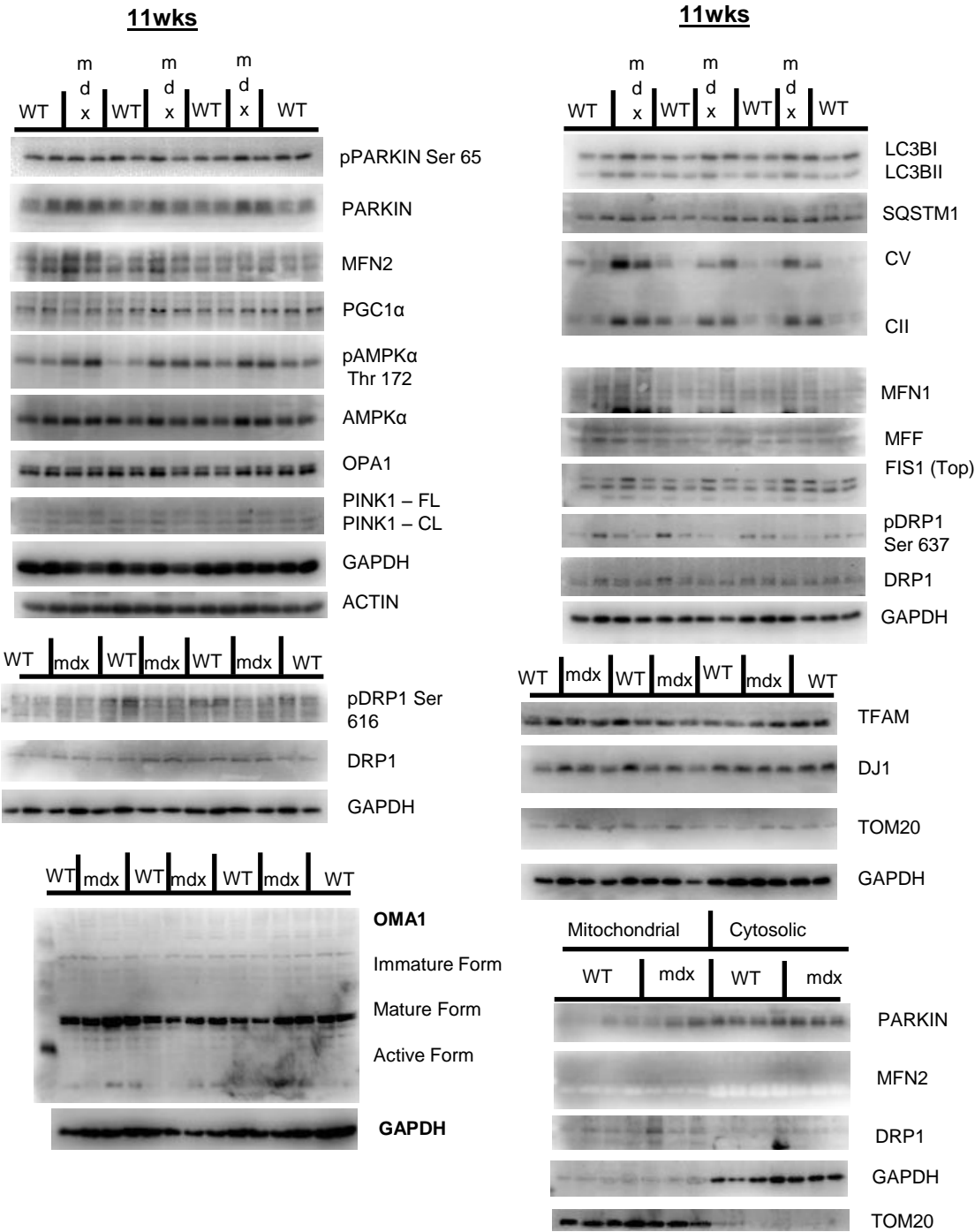

FIGURE S3

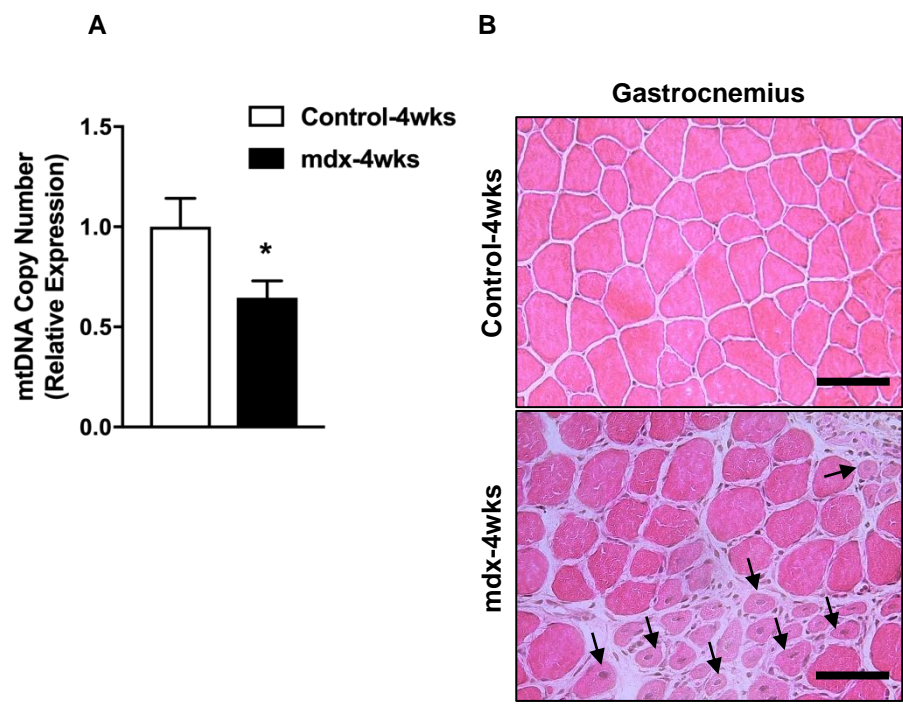

FIGURE S4

A

Control-2wks #1

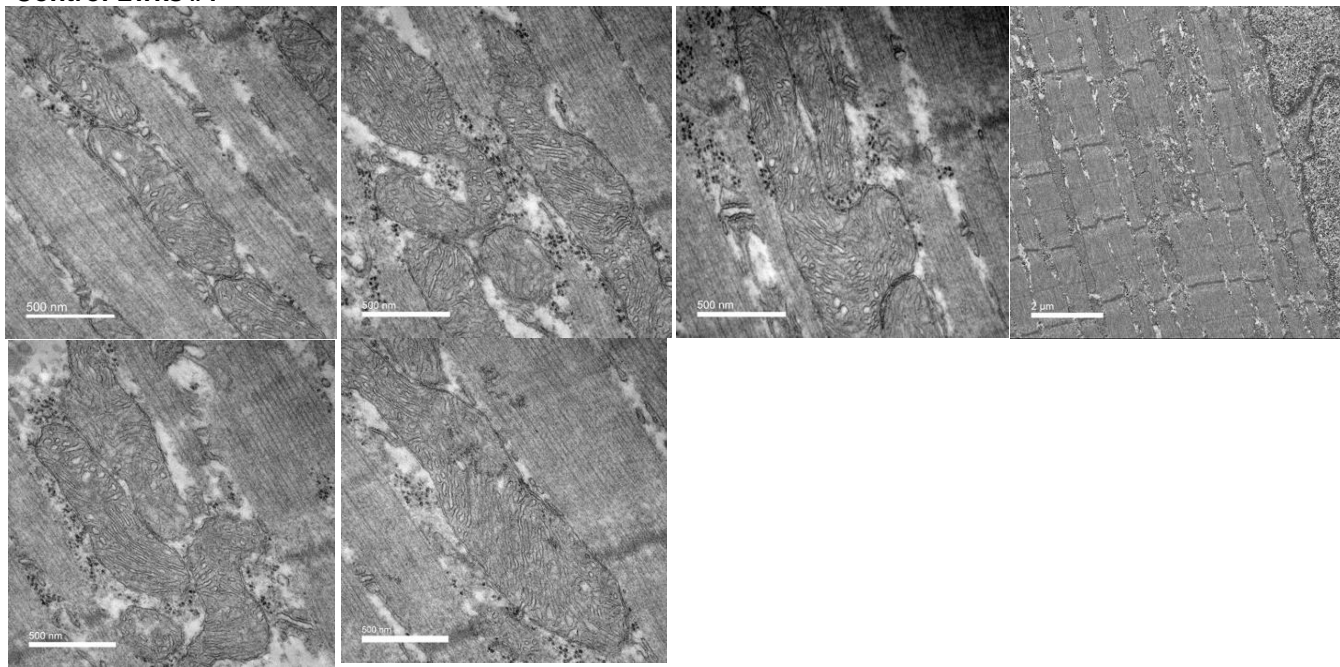

Control-2wks #2

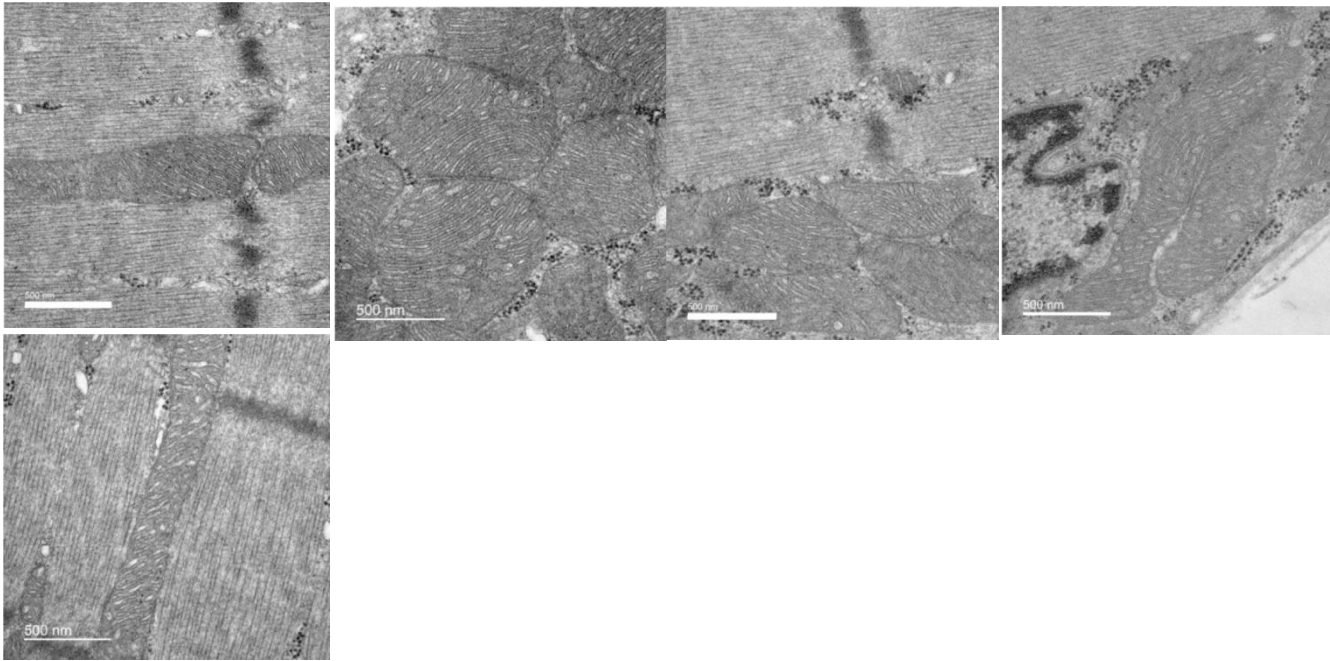

mdx-2wks #1

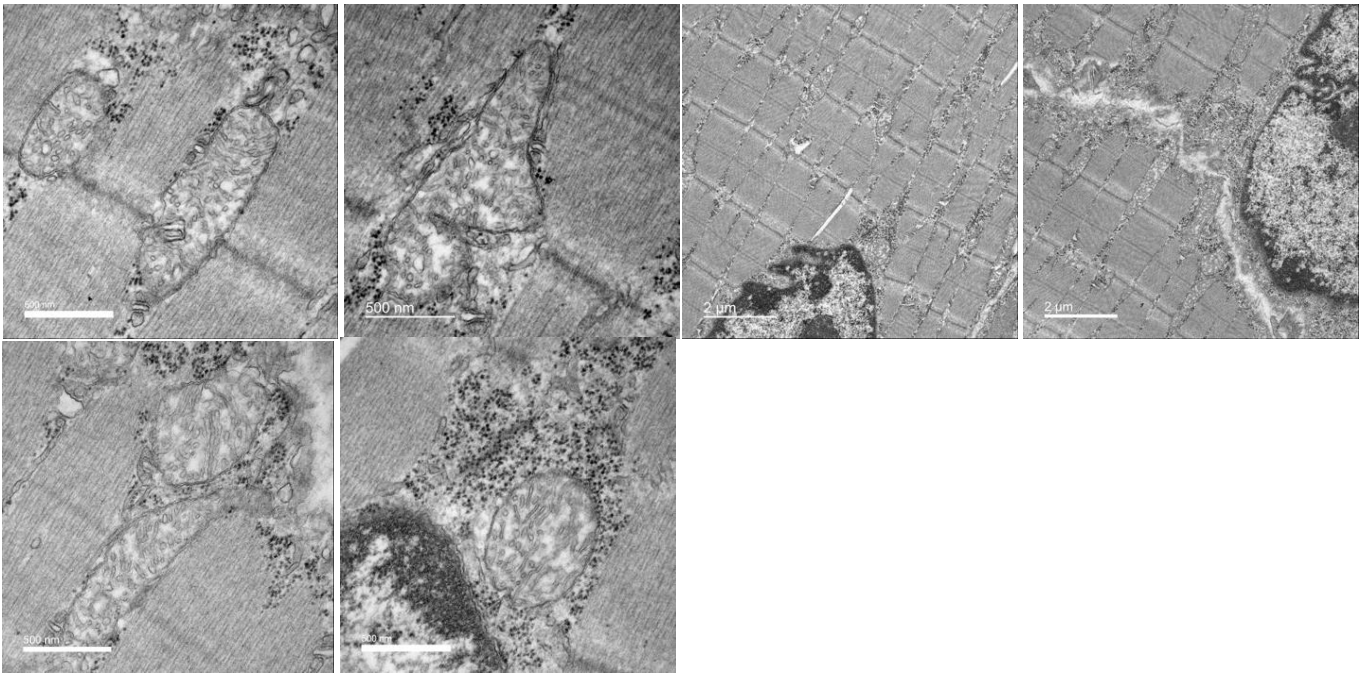

mdx-2wks #2

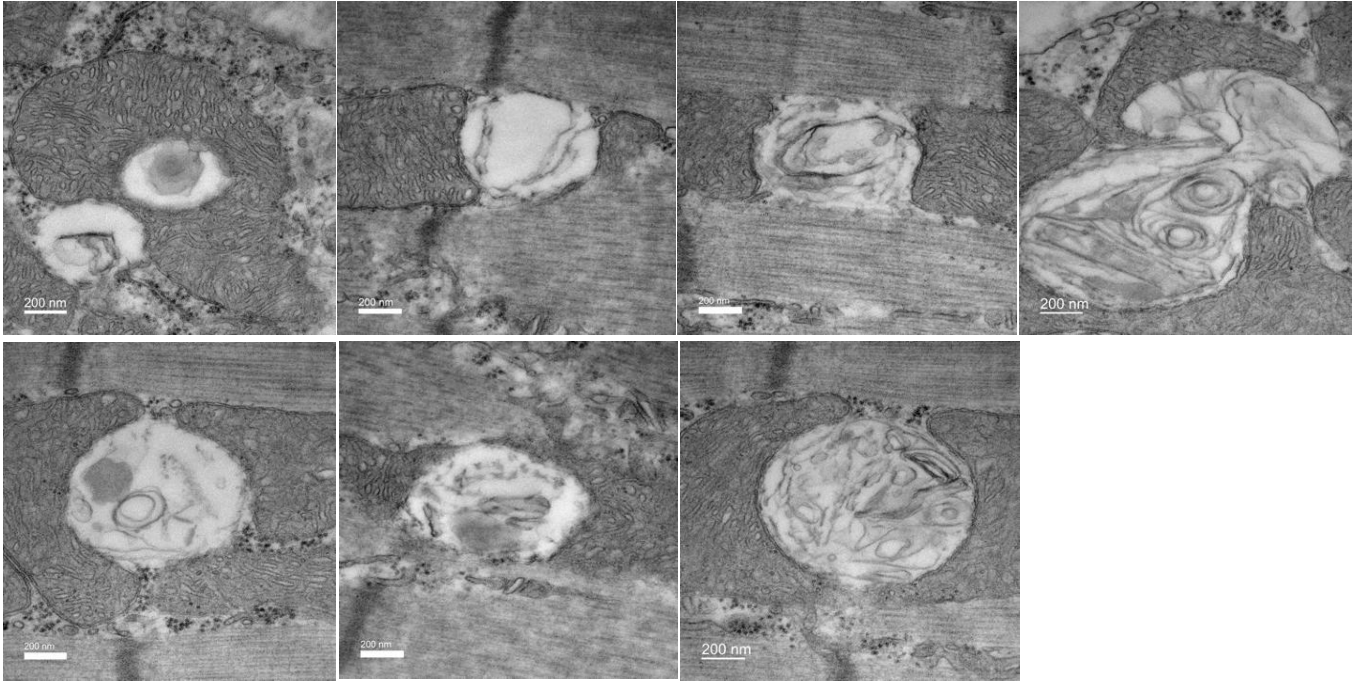

FIGURE S5

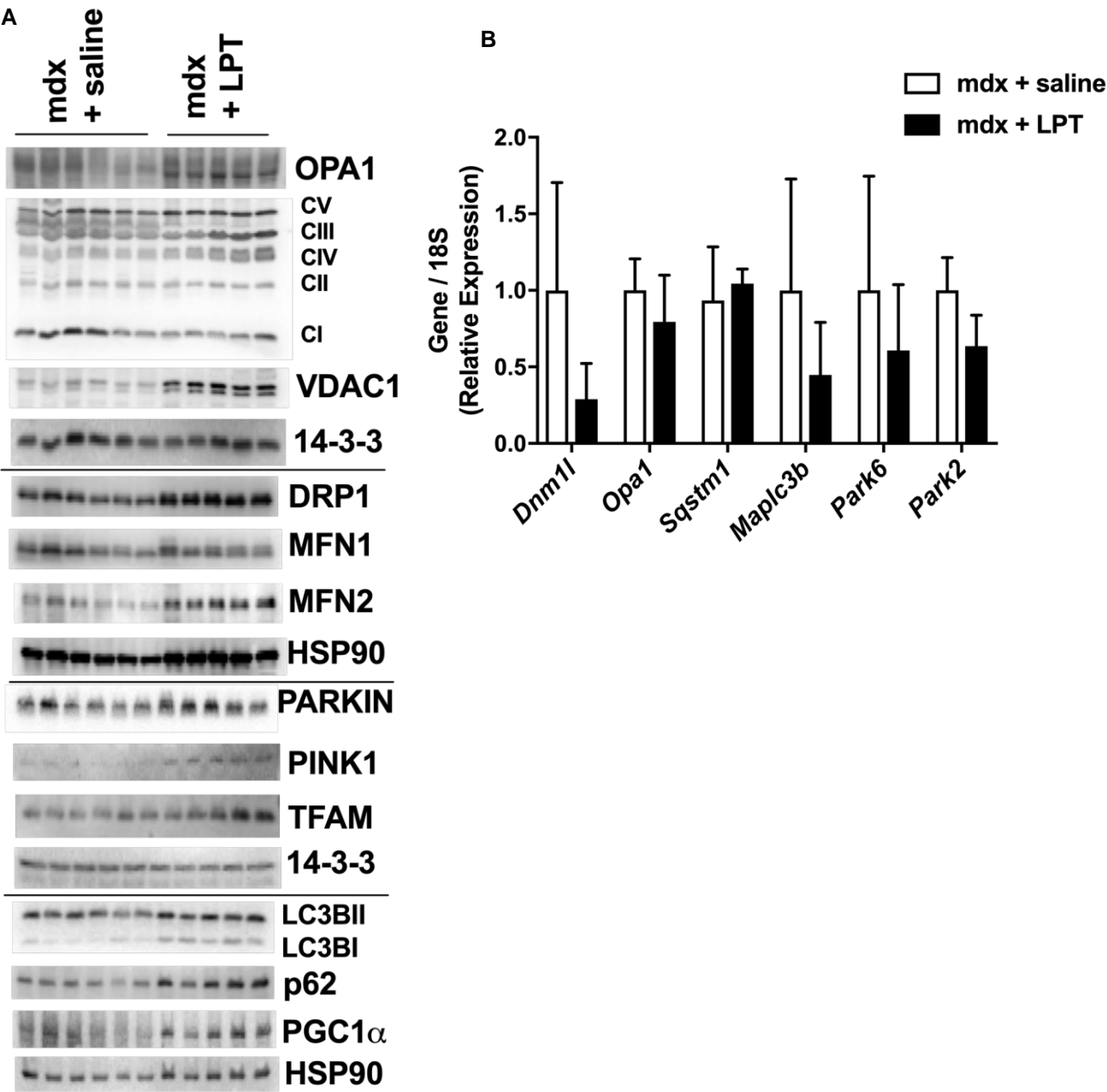

FIGURE S6

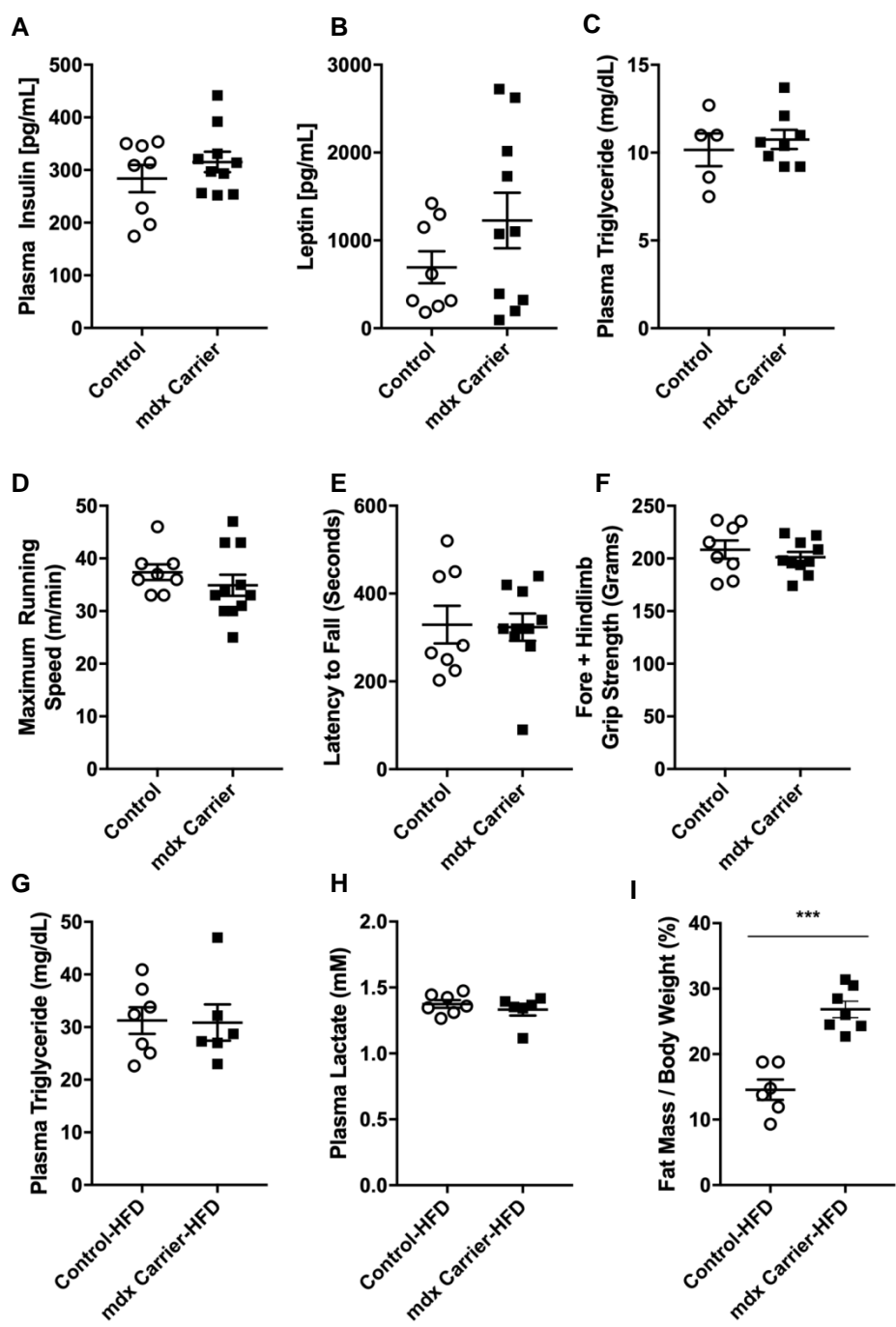

Supplement: FIGURE S1 — Immunoblot images from 40-week-old gastrocnemius muscle. [file Data_Sheet_1.pdf]
